# Atomistic Simulation of Microstructural Evolution of Ni_50.8_Ti Wires during Torsion Deformation

**DOI:** 10.3390/ma15010092

**Published:** 2021-12-23

**Authors:** Shan Liu, Yao Lin, Tao Wu, Guangchun Wang

**Affiliations:** Key Laboratory for Liquid-Solid Structural Evolution and Processing of Materials, Ministry of Education, Shandong University, Jinan 250061, China; liushan890925@163.com (S.L.); linyaoqcs@163.com (Y.L.)

**Keywords:** nitinol shape memory alloy, atomic torsion simulation, martensitic transformation, dislocation

## Abstract

To explore the microstructural evolution of Ni_50.8_Ti wires during torsion deformation, single and polycrystalline models with various grain sizes (*d* = 9 nm, 5.6 nm, and 3.4 nm) were established on an atomic scale to explore their grain morphology evolution, stress-induced martensitic transformation, and dislocation movement. The results indicated that the grains were rotated and elongated to form long strips of grains during the torsion simulation. With the increase in torsion deformation, the elongated grains were further split, forming smaller grains. Stress-induced martensitic transformation took place and the martensite preferentially nucleated near the grain boundary, resulting in the formation of 30% austenites and 50% martensites. Additionally, a certain number of dislocations were generated during the torsion simulation. Under a low degree of torsion deformation, the main mechanism of plastic deformation was dislocation movement, while with a large degree of torsion deformation, the main mechanism of plastic deformation was grain rotation.

## 1. Introduction

NiTi shape memory alloy, as a new functional material that integrates sensing and driving [1,2], has been widely used in aerospace, mechanical engineering, biomedicine, and other fields [3,4]. The shape memory effect of NiTi alloys is determined by thermal-induced martensitic transformation and its reverse transformation during cooling and heating procedures [5]. The essence of super-elasticity lies in stress-induced martensitic transformation and its reverse transformation during loading and unloading processes [6]. This characteristic behavior occurs in parent austenite [7]. When the temperature is above *A*_f_ (the final temperature of austenite transformation), martensite is unstable, and thus, stress-induced martensitic reverse transformation occurs upon unloading. When the temperature is lower than *A*_s_ (the starting temperature of austenite transformation), martensite is stable. If unloaded, a deformed state is maintained in the NiTi alloy, and it can be recovered via the reverse martensitic transformation, but only when the temperature is higher than *A*_f_.

Studies have shown that improving the slip critical stress of the matrix is a key factor for improving the functional stability of NiTi alloys. Methods for improving the matrix slip critical stress mainly include plastic deformation [8], solution aging [9], electric pulse treatment [10], surface shot peening [11], and so on. The mechanisms of these processes are attributed to grain refinement strengthening and precipitation strengthening [12]. For NiTi alloys with a fixed amount of Ni, the maximum ability of the precipitation strengthening process can be determined. Grain refinement strengthening is usually achieved through different severe plastic deformation methods, such as high pressure torsion [13], equal channel angular pressing [14], unidirectional tension/compression [15], stretching–bending [16], and other deformation processes. Thus, the various size grains are obtained via different deformation methods that improve the functional stability of NiTi alloys to different degrees.

However, in the processing and manufacturing of NiTi alloys, thin sheets and filaments are generally used with a thickness or diameter of 0.1–5 mm. Keeping the shape and dimension of the NiTi wires unchanged, the aforementioned plastic deformation methods are restricted to a certain extent due to the geometric size of the material, and are limited to drawing processes [17] such as cold and hot drawing. Therefore, it is necessary to develop a plastic deformation method that is particularly suitable for NiTi wires, in order to improve their functional stability.

Martensitic transformation, including martensite nucleation and growth, occurs during the plastic deformation process, when the external stress of the NiTi alloy reaches the critical stress level of the stress-induced martensitic transformation [18,19]. The grain boundaries, as important features of the phase transformation nucleus, play a significant role in martensitic transformation and its reverse transformation [20]. The smaller the grain size, the higher the ratio of grain boundaries, and thus, the more conducive the process is to the stress-induced martensitic deformation nucleus during loading, and the more beneficial is its reverse phase transformation back to the austenites following unloading. The accumulative residual strain generated in this situation is small, and a good function stability is ultimately obtained [10,16]. Additionally, smaller grains are more conducive to improving the compatible deformation capability during plastic deformation. When the diameter of the NiTi wires is reduced to a certain extent, a small number of grains exist along the radial direction, which can affect the flow stress and strain during the plastic deformation and further influence the stress-induced martensitic transformation. Therefore, it is necessary to carry out related research on the microstructural evolution and mechanical properties of NiTi wires with small dimensions during plastic deformation.

Moreover, the martensitic transformation speed is so fast that current experiments cannot output information on microstructural evolution and atomic force in real time. With the rapid development of modern computer software and hardware technologies, molecular dynamics (MD) simulations have become another method for understanding and recognizing NiTi alloys on the atomic scale as well as experiments and theories [21,22,23,24]. Moreover, the geometric size of NiTi wires with small dimensions further increases the difficulty of plastic deformation. Therefore, MD simulations can be used to conduct relevant research into NiTi wires with small dimensions during plastic deformation. Wang et al. [18] studied an MD simulation of the cyclic degradation of a nanocrystalline NiTi alloy under thermomechanical-coupled cyclic loading conditions. The results showed that the accumulation of plastic deformation is caused by the interface migration and dislocation slip of martensite variants. The main factors of the superelastic degradation observed by Ko et al. [25] under cyclic loading were the accumulated plastic deformation and the residual martensites present, which originated from the synergistic effect of the amorphous and crystalline regions. Due to the presence of the amorphous phase, increases in the stability of the martensite phase cause a sudden decrease in the stress plateau and hysteresis under cyclic loading. In addition, Wu et al. [26] studied the existence of pores in porous NiTi alloys under impact, leading to martensite transformation as well as plastic deformation caused by the transformation.

Summarily, the main issues with NiTi wires are as follows: (i) There are few deformation modes, as the current methods are mainly limited to drawing deformation [17,27]; (ii) There is a lack of related research into the microstructural evolution and mechanical properties of NiTi wires with small dimensions during plastic deformation [27]; and (iii) It is hard to observe and output their microstructural evolution in real time during plastic deformation especially during stress-induced martensitic transformation [28]. Therefore, aiming at Ni_50.8_Ti wires with small dimensions, MD was used here to simulate their unidirectional torsion deformation, to further explore the effects of torsion deformation on the microstructure of NiTi wires with different grain sizes.

## 2. Methodology

**Figure 1:**
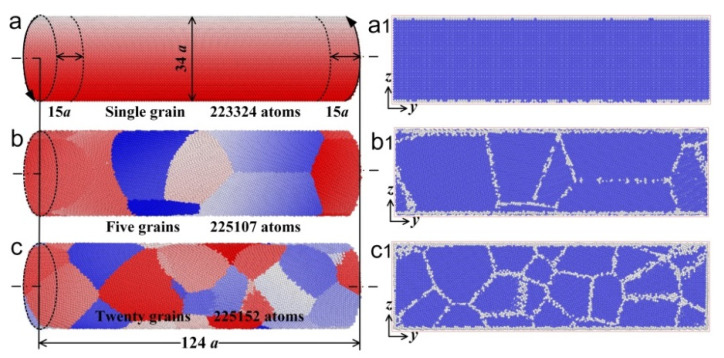
The Ni_50.8_Ti cylinder models and the longitudinal section slices. (**a**) Single crystal cylinder model, containing 223,324 atoms; (**b**) Polycrystalline cylinder model, containing 5 grains and 225,107 atoms; (**c**) Polycrystalline cylinder model, containing 20 grains and 225,152 atoms; (**a1**–**c1**) The longitudinal section slices of the model shown in Figure 1a–c, respectively.

**Figure 2:**
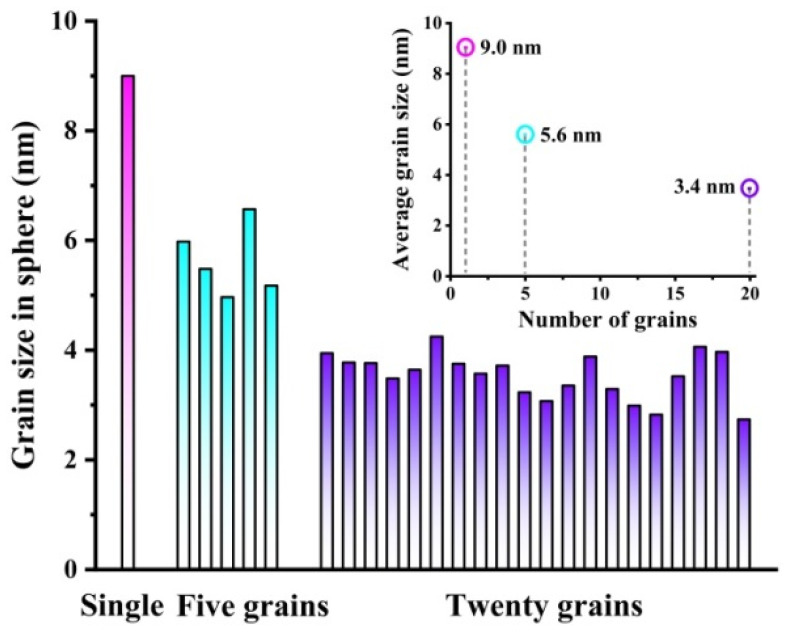
The distribution of specific grain sizes in different Ni_50.8_Ti cylinder models. Each grain is approximately regarded as a sphere. The pink bar graph represents the grain size of the single crystal model, with an average value of 9.0 nm; The blue bar graph represents the grain size of the polycrystalline model with 5 grains, and the average grain size is 5.6 nm; The purple bar graph represents the grain size of the polycrystalline model with 20 grains, and the average grain size is 3.4 nm.

Shape memory alloys with a nominal composition of 50.8 Ni (at. %) are widely used in the engineering and biomedical fields. The chemical compositions of the selected Ni_50.8_Ti wires are listed in Table 1. In order to explore the microstructural evolution of Ni_50.8_Ti wires with different grain sizes during unidirectional torsion deformation, MD simulations were carried out by a large-scale atomic/molecular massively parallel simulator (LAMMPS), which was based on the second nearest neighbor modified embedded-atom potential (2NN-MEAM) [29]. The simulation box was a 101.966 × 371.876 × 101.966 Å^3^ cube with a lattice constant *a* of 2.999 Å. The x, y, and z directions, respectively, corresponded to [100], [010], and [001] orientations. Non-periodic and shrink-wrapped boundary conditions were used in all three directions. The simulation step was 0.001 ps, and the cutoff radius was 5 Å. Three cylinder models of Ni_50.8_Ti wires with different grain sizes were built via the Voronoi method. A single crystal cylinder model in Figure 1a and two polycrystalline cylinder models containing 5 and 20 grains in Figure 1b,c were, respectively, established and made up of ~220,000 atoms with a size of *ϕ* 34*a* × 124*a*. As the grains were regarded as spheres, the average grain sizes of the cylinder models shown in Figure 1a–c were 9 nm, 5.6 nm, and 3.4 nm, respectively. The specific grain size in different models is shown in Figure 2. Combined with Table 2, a scale parameter *N* is introduced, indicating the number of grains along the diameter of the wires or the thickness of the sheets. The parameter *N* can be expressed as *N* = *D*/*d*, where *D* in the current work is the diameter of the cylinder model and *d* is the average grain size of the cylinder model. With a constant model size and an increasing number of grains, the average grain size decreased, and the value of the scale parameter increased.

As the temperature *A*_f_ predicted by the 2NN-MEAM potential function was below 25 °C, the simulation temperature had to be higher than *A*_f_ to ensure the material would be in a B2 austenite state. Therefore, the subsequent simulations were all carried out at 25 °C (>*A*_f_). The conjugate gradient method was applied to minimize the energy of the model, and then the model was fully relaxed at a given temperature of 25 °C for 100 ps. A Nosé–Hoover thermostat and a barostat were used to control the temperature and pressure, respectively. During the thermal equilibrium stage and torsion deformation, the MD simulations were performed in a canonical ensemble (NVT). In the torsion simulation process, the length of the clamping ends on the left and right sides of the cylinder model was set as 44.985 Å, namely 15 *a*. The torsion speed applied to the clamping ends was ±5 × 10^−3^ r/ps, and then all the torsion simulations of the NiTi models with different grain sizes were run at 800 ps.

The simulation results were visualized and analyzed by OVITO software. A dislocation analysis (DXA) module was used for dislocation analysis. The magenta lines correspond to dislocations of the 1/3<1$\bar{1}$00> type, the orange lines to dislocations of the <1$\bar{1}$00> type, and the blue lines to dislocations of other types that could not be recognized by OVITO. A polyhedral template matching (PTM) module was applied to analyze the structure evolution, where the blue atoms correspond to austenites (B2), the red atoms to martensites (B19′) [22,30,31], the green atoms to highly stressed austenites [30], and the white atoms to other structures that could not be recognized by OVITO. A Python script module assisted in obtaining the content *α* of each phase via script file.

## 3. Results and Discussion

### 3.1. Evolution of Grain Morphology

**Figure 3:**
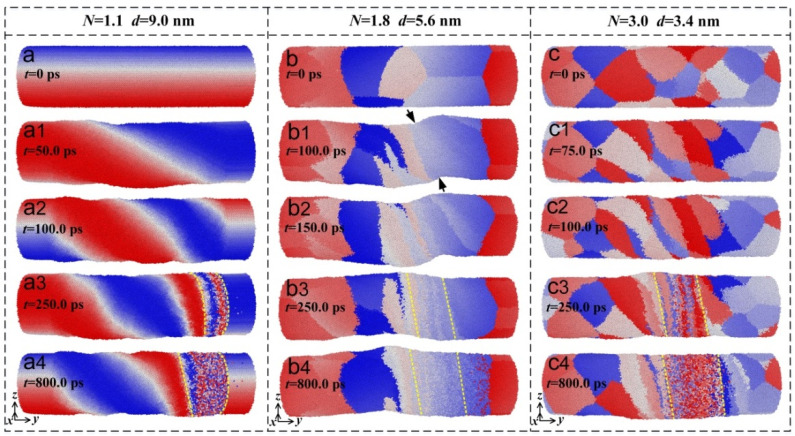
During the torsion simulation, the evolution of the grain morphology in different models at various times. (**a**–**a4**) Single crystal model with an average grain size of 9.0 nm. (**a**) *t* = 0 ps; (**a1**) *t* = 50.0 ps; (**a2**) *t* = 100.0 ps; (**a3**) *t* = 250.0 ps; (**a4**) *t* = 800.0 ps. (**b**–**b4**) Polycrystalline model with an average grain size of 5.6 nm. (**b**) *t* = 0 ps; (**b1**) *t* = 100.0 ps; (**b2**) *t* = 150.0 ps; (**b3**) *t* = 250.0 ps; (**b4**) *t* = 800.0 ps. (**c**–**c4**) Polycrystalline model with an average grain size of 3.4 nm. (**c**) *t* = 0 ps; (**c1**) *t* = 75.0 ps; (**c2**) *t* = 100.0 ps; (**c3**) *t* = 250.0 ps; (**c4**) *t* = 800.0 ps.

During the torsion simulation, there was a great difference in the grain evolution at various times even in the same model, as shown in Figure 3. Figure 3a–c indicates that at the initial state of *t* = 0, the average grain size decreased with the increase in scale parameter *N* during the torsion simulation. When the simulation time increased, the grains were rotated and then elongated, resulting in the change of the equiaxed grains from their initial state to become long strips of grains, as shown in Figure 3(a1,a2); Figure 3(b1,b2); and Figure 3(c1,c2). When the simulation time further increased, the amount of torsion deformation increased, and thus, part of the elongated grains were spilt to form much smaller grains as indicated in Figure 3(b3,c3). As the simulation time was furthermore prolonged, large amounts of torsion deformation were caused and the atoms moved over a long distance, showing a disordered state and then exhibiting local amorphous characteristics [22] as shown in Figure 3(a4–c4).

However, there also existed a distinction between the grain evolution at the same time in different models. At the simulation time of *t* = 100.0 ps, the degree of grain deformation varied in different models with the increase in the scale parameter. At *t* = 250.0 ps, various degrees of large deformation were located in different positions in the models. As the simulation time was prolonged to 800.0 ps, different amounts of amorphous structures were generated in different models. Additionally, when the model contained a small number of grains, the size of each grain varied in the model, and it changed over a relatively wide range resulting in poorly compatible deformation capabilities. The cross section of the cylinder model was relatively misaligned, and bent to a certain extent along the axis of the model as shown by the arrow in Figure 3(b1).

### 3.2. Martensitic Transformation

**Figure 4:**
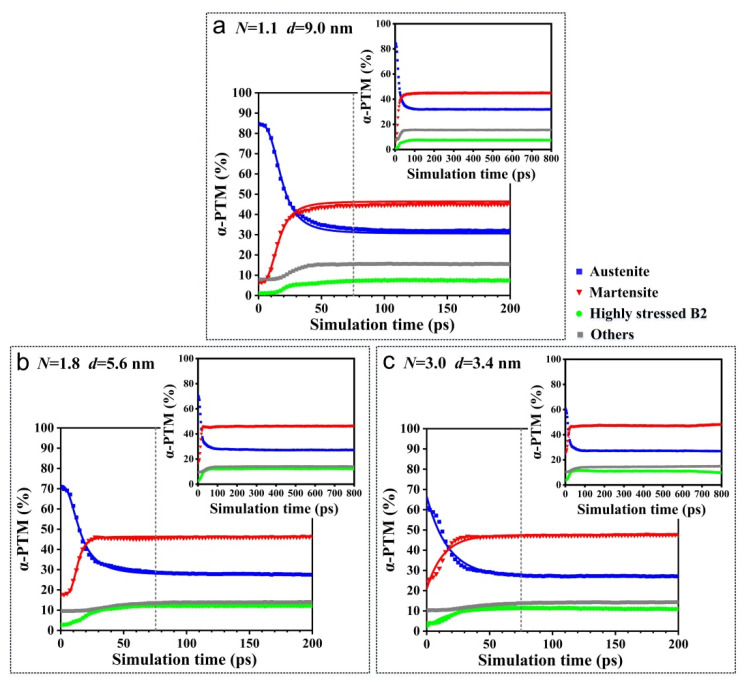
The relationship between the phase content and simulation time in different models with various grain sizes during torsion deformation. (**a**) Single crystal model with an average grain size of 9.0 nm; (**b**) Polycrystalline model with an average grain size of 5.6 nm; (**c**) Polycrystalline model with an average grain size of 3.4 nm.

During the torsion simulation, the stress-induced martensitic transformation took place when the amount of torsion deformation increased. Various sizes of grains were distributed across the different models. Figure 4 shows the content of each phase generated during the martensitic transformation; it indicates that at the initial stage of torsion simulation (*t* = 0 ps), the austenite phase content gradually decreased according to the increase in the average grain size. This is because different numbers of grains are contained in different models. Under the increase of the scale parameter, the proportion of grain boundaries increased. The disordered arrangement of atoms on the grain boundaries was classified as an ‘other structure’ type by the PTM algorithm, resulting in a relative decrease in the initial austenite phase content. In the same model, as the simulation time increased, the stress-induced martensitic transformation occurred, resulting in a decrease in the austenite phase content and an increase in the martensite phase content. As the simulation time exceeded 75.0 ps, the phase content no longer changed significantly, as shown in the inset in Figure 4a–c. Additionally, in different models with various scale parameters and various average grain sizes, the obtained phase contents of the austenite and martensite were basically the same, at 30% and 50%, respectively. This indicates that the scale parameter value had no significant effect on the phase contents of the austenite and martensite formed during the martensitic transformation. This is because as the simulation time increased, the amount of torsion deformation increased. Under the shear stress conditions, the stress value in the different models all reached a stress level that induced the martensitic transformation, and thus, the different models all underwent complete martensitic transformation resulting in the same phase contents of austenite and martensite.

**Figure 5:**
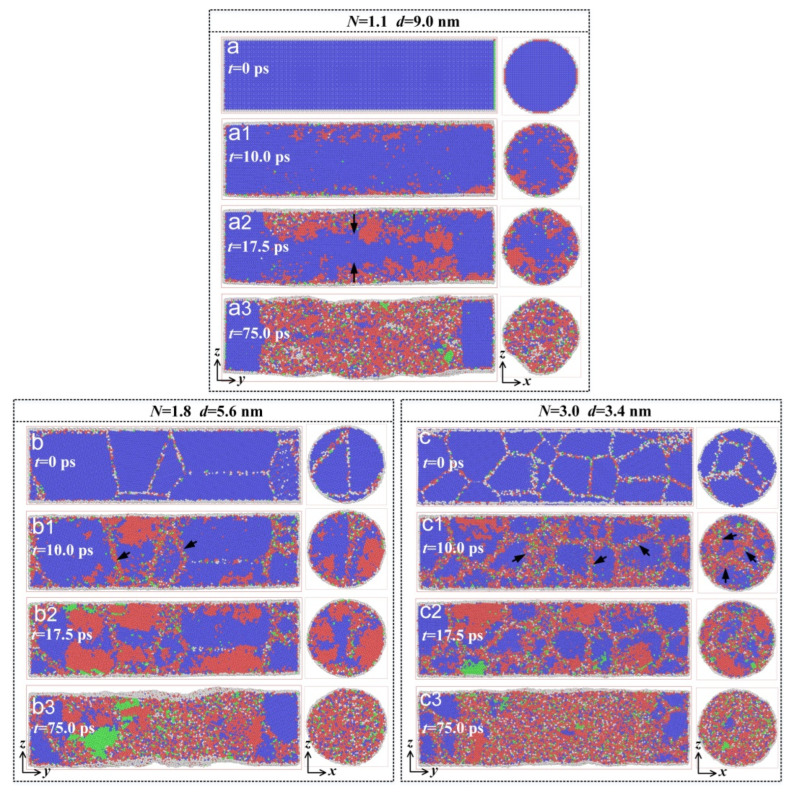
The microstructural evolution of different models at various times during the torsion simulation. (**a**) Single crystal model with an average grain size of 9.0 nm, *t* = 0 ps; (**a1**) *t* = 10.0 ps; (**a2**) *t* = 17.5 ps; (**a3**) *t* = 75.0 ps. (**b**) Polycrystalline model with an average grain size of 5.6 nm, *t* = 0 ps; (**b1**) *t* = 10.0 ps; (**b2**) *t* = 17.5 ps; (**b3**) *t* = 75.0 ps. (**c**) Polycrystalline model with an average grain size of 3.4 nm, *t* = 0 ps; (**c1**) *t* = 10.0 ps; (**c2**) *t* = 17.5 ps; (**c3**) *t* = 75.0 ps. The blue atoms correspond to austenites, the red atoms to martensites, the green atoms to highly stressed austenites, and the white atoms to other structures that cannot be recognized by OVITO.

Figure 5 shows the microstructural evolution in the models with various grain sizes during the torsion simulation; it indicates that in the simulation initial stage, the nucleation of the stress-induced martensitic transformation preferentially took place near the grain boundary, and then expanded from the grain boundary region to the grain interior region as shown in Figure 5(a1–c1). With the increase in simulation time, the stress-induced martensitic transformation spread from the circumference to the center, as indicated in Figure 5(a2–c2). This is because the shear stress gradually increased along the radial direction and reached the maximum value at the circumference, resulting in the extension of the martensitic transformation from the circumference to the center during the early stage of the torsion deformation. When the simulation time further increased, martensitic transformation occurred throughout the entire model, forming large amounts of martensites as shown in Figure 5(a3–c3). Additionally, the martensite phase content formed in the polycrystalline model was higher than that of the single crystal model under the same torsion deformation conditions, as shown in Figure 5(a1–c1), which further demonstrates that the existence of the grain boundary is conducive to the nucleation of martensitic transformation [20].

### 3.3. Evolution of Dislocation

**Figure 6:**
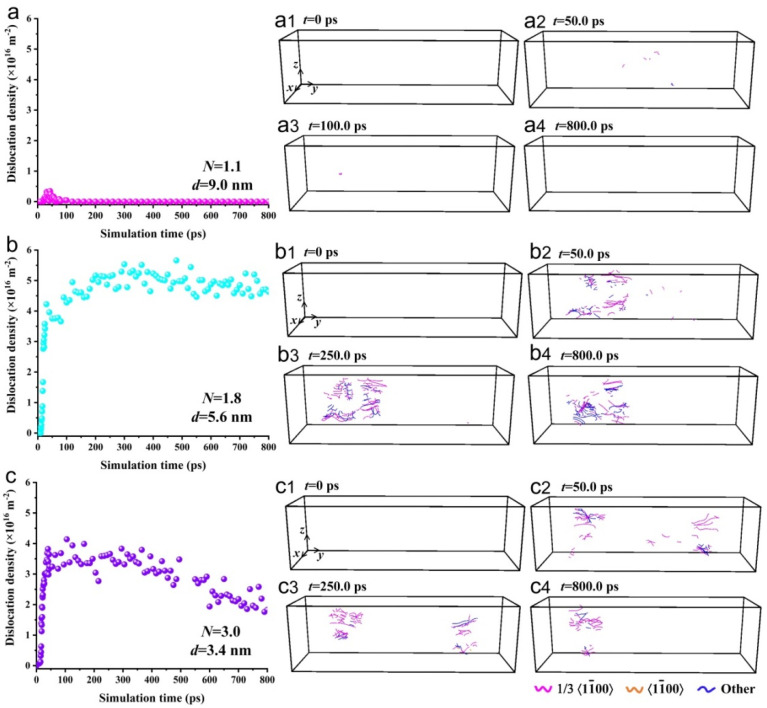
The relationship between the dislocation density and simulation time, as well as the dislocation evolution during the torsion simulation. (**a**–**a4**) Single crystal model with an average grain size of 9.0 nm; (**a1**) *t* = 0 ps; (**a2**) *t* = 50.0 ps; (**a3**) *t* = 100.0 ps; (**a4**) *t* = 800.0 ps. (**b**–**b4**) Polycrystalline model with an average grain size of 5.6 nm; (**b1**) *t* = 0 ps; (**b2**) *t* = 50.0 ps; (**b3**) *t* = 250.0 ps; (**b4**) *t* = 800.0 ps. (**c**–**c4**) Polycrystalline model with an average grain size of 3.4 nm; (**c1**) *t* = 0 ps; (**c2**) *t* = 50.0 ps; (**c3**) *t* = 250.0 ps; (**c4**) *t* = 800.0 ps. The magenta lines correspond to dislocations of the 1/3<1$\bar{1}$00> type, the orange lines to dislocations of the <1$\bar{1}$00> type, and the blue lines to dislocations of other types that cannot be recognized by OVITO.

Figure 6 shows the dislocation evolution in the different models during the torsion simulation; it indicates that as the simulation time increased, the amount of deformation increased, forming a relatively small number of dislocations in the single crystal model as shown in Figure 6a. As the scale parameter increased, the average grain size decreased, and the number of dislocations in the polycrystalline model increased significantly. The average dislocation density was much higher than the experimental value of 10^10^ m^−2^ in the metals that were subjected to the full annealing treatment, and reached the same order of magnitude (10^16^ m^−2^) as that generated in the metals after severe deformation [20], as shown in Figure 6b,c.

**Figure 7:**
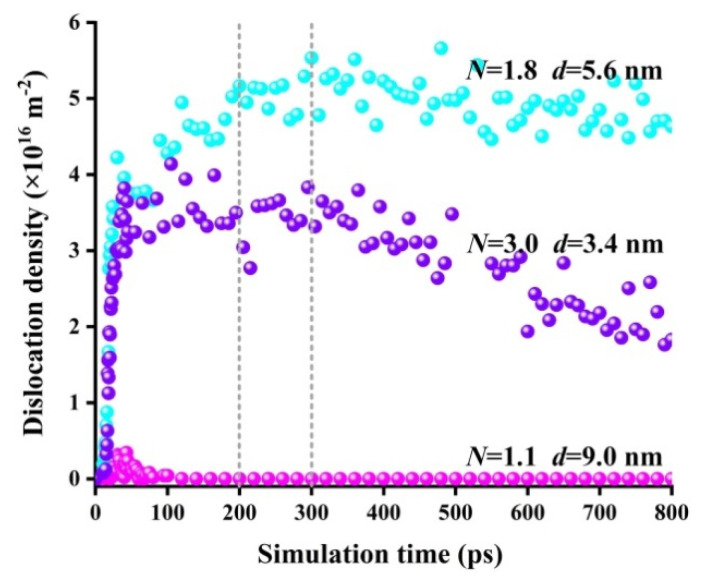
The relationship between the simulation time and the dislocation density in different models during the torsion simulation.

Combined with Figure 7, this indicates that for the polycrystalline model with a small amount of torsion deformation, the average dislocation density increased as the simulation time increased (*t* < 200.0 ps). The main mechanism of the torsion deformation was dislocation movement. Under a large amount of torsion deformation, the dislocation density changed differently as the simulation time further increased (*t* > 300.0 ps). Both the average grain size and dislocation density decreased with increases in the scale parameter; this is because small grains are more conducive to coordinating the plastic deformation of NiTi alloys to a certain extent [32]. The proportion of grain boundaries increased with the decrease in grain size. The dislocations moved to the vicinity of the grain boundaries and then spread no farther. At this time, the dislocation movement was supplemented by the grain rotation. This indicates that with large amounts of torsion deformation, the main mechanism of torsion deformation is grain rotation.

**Figure 8:**
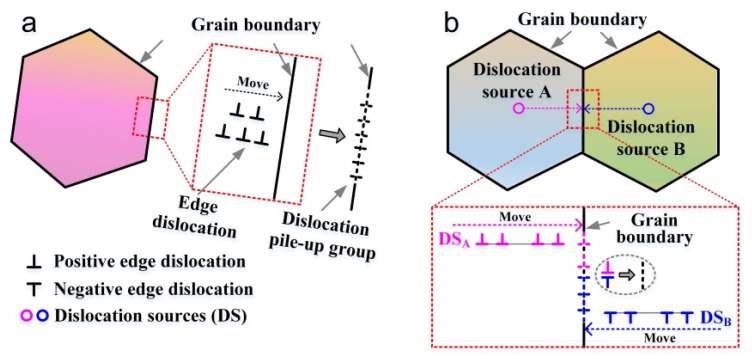
Schematic diagram of dislocations absorbed by the grain boundary. (**a**) Edge dislocations accumulate before the grain boundary, and may be absorbed by the grain boundary, forming dislocation pile-up groups. (**b**) Dislocation sources (DS_A_ and DS_B_) from the center of two adjacent grains emit edge dislocations with opposite signs, which are absorbed by the grain boundaries after their accumulation in the grain boundaries. These grain boundary dislocations with opposite signs may cancel each other out.

Regarding the two polycrystalline models, the average dislocation density decreased according to the decrease in grain size. This may be attributed to the following reasons: (*i*) Grain boundaries hinder the movement of dislocations [11,33]. The finer the grains, the higher the ratio of the grain boundaries and the greater their hindering effects. Moreover, in the large grain-size model, the relative distance between the intragranular dislocation and the grain boundary was greater than that in the small-grain-size-model. During the dislocation movement, the high ratio of the grain boundary had a greater hindering effect on the dislocation. (*ii*) The smaller the grain size, to a certain extent, the better the coordinated plastic deformation ability [32]. Under the same strain conditions, more dislocations need to be generated in the large grain-size model to coordinate the plastic deformation, while the small grain-size model needed lower numbers of dislocations for plastic deformation to occur. (*iii*) The finer the grain, the higher the grain boundary ratio, and the easier it is to be absorbed by the grain boundaries [7,20], forming dislocation pile-up groups in the grain boundaries as shown in Figure 8a. Alternatively, the other case is that dislocation sources from the center of two adjacent grains emit edge dislocations with opposite signs, which are also absorbed by the grain boundaries after their accumulation in the grain boundaries as shown in Figure 8b. Based on these two cases, the dislocation density is ultimately reduced under the same dislocation levels. However, for the average dislocation density generated in a single crystal, the differences and connections between single crystals and polycrystals still need to be further explored.

## 4. Conclusions

To explore the microstructural evolution of Ni_50.8_Ti wires during unidirectional torsion deformation, single crystal and polycrystalline models with various grain sizes (*d* = 9 nm, 5.6 nm, and 3.4 nm) were established on an atomic scale to explore the grain morphology evolution, stress-induced martensitic transformation and its phase content, and the dislocation movement. The following conclusions could be drawn:

(1) The grains were rotated and elongated to form long strips of grains. When the simulation time increased, the amount of deformation increased, and part of the elongated grains were split, forming smaller grains. Stress-induced martensitic transformation took place during the torsion simulation, resulting in the formation of 30% austenites and 50% martensites.
(2) A number of dislocations were produced during the torsion simulation, and the average dislocation density reached the same order of magnitude that was generated in the metals following severe deformation. With a small amount of torsion deformation, the main plastic deformation mechanism was dislocation movement. With a large amount of torsion deformation, the main mechanism was grain rotation.
(3) Although the model size and simulated torsion speed were different from the parameter ranges of the actual torsion tests, they could still predict the microstructural evolution of the NiTi alloy during the torsion deformation process, which is more conducive to understanding the evolution law of different models with various grain sizes during torsion deformation. It is hoped that an MD model closer to real-life dimensions could be established using real-life parameter settings in the future, which will mainly rely on the computer software/hardware equipment available and its calculation efficiency.

## Figures and Tables

**Table 1:** The chemical compositions of the Ni_50.8_Ti wire.

Element	Main Composition		Impurity Composition						
	Ti	Ni	C	N	O	H	Co	Cu	Cr
wt.%	Remainder	55.88	0.012	0.002	0.040	0.001	<0.046		
at.%	Remainder	50.8	0.05	0.008	0.130	0.053	<0.045		

**Table 2:** The diameter *D*, average grain size *d*, and scale parameter *N* in different NiTi models.

Number of Grains	1	5	20
Diameter of model *D*: (nm)	10.1966		
Average grain size *d*: (nm)	9.0	5.6	3.4
*N* = *D*/*d*	1.1	1.8	3.0

Note: *D* is the diameter of the cylinder model, *d* is the average grain size of the model, and *N* is the parameter expressed as *N* = *D*/*d*.

## Data Availability

The data provided in this study could be released upon reasonable request.

## References

[1] Mao Z., Xu Z., Wang Q. (2020). Shape memory alloy actuator with active cooling device and deflectable winglet application. Smart Mater. Struct..

[2] Karna P., Prabu S., Karthikeyan S.C., Mithun R., Palani I.A. (2020). Investigations on laser actuation and life cycle characteristics of NiTi shape memory alloy bimorph for non-contact functional applications. Sens. Actuators A Phys..

[3] Barbarino S., Pecora R., Lecce L., Concilio A., Ameduri S., De Rosa L. (2011). Airfoil Structural Morphing Based on S.M.A. Actuator Series: Numerical and Experimental Studies. J. Intell. Mater. Syst. Struct..

[4] Zhu J., Zeng Q., Fu T. (2019). An updated review on TiNi alloy for biomedical applications. Corros. Rev..

[5] Kim H.Y., Ikehara Y., Kim J.I., Hosoda H., Miyazaki S. (2006). Martensitic transformation, shape memory effect and superelasticity of Ti–Nb binary alloys. Acta Mater..

[6] Zhao T., Kang G. (2022). Experimental study and life prediction on fatigue failure of NiTi shape memory alloy under multi-axial one-way shape memory cyclic loadings. Int. J. Fatigue.

[7] Otsuka K., Ren X. (2005). Physical metallurgy of Ti–Ni-based shape memory alloys. Prog. Mater. Sci..

[8] Lim Y.G., Han S.H., Choi E., Kim W.J. (2018). Shape memory and superelasticity of nanograined Ti-51.2 at.% Ni alloy processed by severe plastic deformation via high-ratio differential speed rolling. Mater. Charact..

[9] Liu S., Lin Y., Wang G., Wang X. (2021). Effect of varisized Ni4Ti3 precipitate on the phase transformation behavior and functional stability of Ti-50.8 at.% Ni alloys. Mater. Charact..

[10] Liu S., Zhu J., Lin X., Wang X., Wang G. (2020). Coupling effect of stretch-bending deformation and electric pulse treatment on phase transformation behavior and superelasticity of a Ti-50.8at.% Ni alloy. Mater. Sci. Eng. A.

[11] Li X., Chen H., Guo W., Guan Y., Wang X. (2021). Improved superelastic stability of NiTi shape memory alloys through surface nano-crystallization followed by low temperature aging treatment. Intermetallics.

[12] Wang X., Kustov S., Li K., Schryvers D., Verlinden B., Humbeeck J.V. (2015). Effect of nanoprecipitates on the transformation behavior and functional properties of a Ti–50.8 at.% Ni alloy with micron-sized grains. Acta Mater..

[13] Derakhshandeh M.R., Farvizi M., Javaheri M. (2020). Effects of high-pressure torsion treatment on the microstructural aspects and electrochemical behaviour of austenitic NiTi shape memory alloy. J. Solid State Electrochem..

[14] Yanqiu Z., Shuyong J. (2017). The Mechanism of Inhomogeneous Grain Refinement in a NiTiFe Shape Memory Alloy Subjected to Single-Pass Equal-Channel Angular Extrusion. Metals.

[15] Zhang Y., Jiang S., Tang M., Yan B., Zhao C. (2020). Mechanisms for influence of post-deformation annealing on microstructure of NiTiFe shape memory alloy processed by local canning compression. J. Mater. Processing Technol..

[16] Liu S., Zhu J., Lin Y., Wang G., Wang X. (2021). Effect of stretching-bending deformation and aging treatment on phase transformation behavior and superelasticity of Ti-50.8 at.% Ni alloy. Intermetallics.

[17] Wang T., Ma Z., Rao X., Ren Y., Liu Y., Yu K., Cui L. (2020). Temperature-dependence of superelastic stress in nanocrystalline NiTi with complete transformation capability. Intermetallics.

[18] Wang B., Kang G., Yu C., Gu B., Yuan W. (2021). Molecular dynamics simulations on one-way shape memory effect of nanocrystalline NiTi shape memory alloy and its cyclic degeneration. Int. J. Mech. Sci..

[19] Choi W.S., Pang E.L., Ko W.S., Jun H., Choi P.P. (2021). Orientation-dependent plastic deformation mechanisms and competition with stress-induced phase transformation in microscale NiTi. Acta Mater..

[20] Phule P.P. (2009). Essentials of Material Science and Engineering.

[21] Wang B., Kang G., Wu W., Zhou K., Kan Q., Yu C. (2020). Molecular dynamics simulations on nanocrystalline super-elastic NiTi shape memory alloy by addressing transformation ratchetting and its atomic mechanism. Int. J. Plast..

[22] Zhang Y., Jiang S., Wang M. (2020). Atomistic investigation on superelasticity of NiTi shape memory alloy with complex microstructures based on molecular dynamics simulation. Int. J. Plast..

[23] Liu S., Lin Y., Han L., Wang X., Zhao G., Wang G. (2021). Atomistic simulation of microstructure evolution of NiTi single crystals in bending deformation. Comput. Mater. Sci..

[24] Song Z., Tang X., Chen X., Fu T., Zheng H., Lu S. (2021). Nano-indentation and nano-scratching of pure nickel and NiTi shape memory alloy thin films: An atomic-scale simulation. Thin Solid Film..

[25] Ko W.S., Choi W.S., Xu G., Choi P.P., Ikeda Y., Grabowski B. (2021). Dissecting functional degradation in NiTi shape memory alloys containing amorphous regions via atomistic simulations. Acta Mater..

[26] Wu Z., Chen X., Fu T., Zheng H., Zhao Y. (2021). Molecular Dynamics Investigation of the Influence of Voids on the Impact Mechanical Behavior of NiTi Shape-Memory Alloy. Materials.

[27] Racek J., Duchon J., Vronka M., Cieslar M. (2020). TEM observation of twins in surface grains of superelastic NiTi wire after cyclic loading. Mater. Sci. Eng. A.

[28] Sun D., Jiang S., Zhang Y., Yan B., Yu J. (2021). Influence of annealing on incomplete detwinning and deformation twinning in equiatomic NiTi shape memory alloy undergoing severe plastic deformation. J. Alloy. Compd..

[29] Ko W.S., Grabowski B., Neugebauer J. (2015). Development and application of a Ni-Ti interatomic potential with high predictive accuracy of the martensitic phase transition. Phys. Rev. B.

[30] Lu H.Y., Chen C.H., Tsou N.T. (2018). The Analysis of Superelasticity and Microstructural Evolution in NiTi Single Crystals by Molecular Dynamics. Materials.

[31] Li B., Shen Y., An Q. (2020). Structural origin of reversible martensitic transformation and reversible twinning in NiTi shape memory alloy. Acta Mater..

[32] Tyc O., Molnarova O., Sittner P. (2021). Effect of microstructure on fatigue of superelastic NiTi wires. Int. J. Fatigue.

[33] Yan B., Jiang S., Sun D., Wang M., Yu J., Zhang Y. (2021). Martensite twin formation and mechanical properties of B2 austenite NiTi shape memory alloy undergoing severe plastic deformation and subsequent annealing. Mater. Charact..

